# Evaluation of therapeutic potential of the silver/silver chloride nanoparticles synthesized with the aqueous leaf extract of *Rumex acetosa*

**DOI:** 10.1038/s41598-017-11853-2

**Published:** 2017-09-14

**Authors:** Sobha Kota, Pradeep Dumpala, Ratna Kumari Anantha, Mahendra Kumar Verma, Surendranath Kandepu

**Affiliations:** 1 Department of Biotechnology, R.V.R. & J.C. College of Engineering (A), Guntur, 522 019 Andhra Pradesh India; 2 Centre for Biotechnology, Acharya Nagarjuna University, Nagarjuna Nagar, 522 010 Andhra Pradesh India; 3 Department of Biological Sciences, Indian Institute of Science Education and Research, Bhopal, 462 066 Madhya Pradesh India; 4 Department of Physics, R.V.R. & J.C. College of Engineering (A), Guntur, 522 019 Andhra Pradesh India

## Abstract

Silver nanoparticles were green synthesized with the aqueous leaf extract of the widely consumed green leafy vegetable, *Rumex acetosa* (sorrel) and the obtained silver nanoparticles (Ag NPs) were tested for their *in vitro* antioxidant potential, cytotoxicity against human osteosarcoma (HOS) cell lines and antibacterial effects against sixteen human pathogenic clinical isolates. Different analytical techniques viz. UV-vis, FTIR, XRD, SEM-EDX and TEM were employed to characterize the synthesized Ag NPs. Surface Plasmon spectra for the Ag NPs with brownish black color were centered approximately at 448 nm. FTIR analysis revealed the presence of reactive N-H and O-H groups that are effective in reducing Ag(I) ions to Ag(0) which then reacted with the contents of the extract to AgCl/Ag_2_C_2_O_4_. From SEM and TEM analyses, the particles were found to be predominantly spherical in shape and ranged in size from 5 nm to 80 nm, but were largely in the range of 15 nm to 20 nm. Ag NPs showed considerable antioxidant activity, and all the sixteen clinical isolates of human pathogens tested were significantly inhibited. Also, HOS cell lines were significantly (p < 0.05) inhibited at 25% concentration of the Ag NPs extract, while showing a marginal revival at 50% and 100% concentrations.

## Introduction

Design, synthesis and characterization of non-toxic bio-active materials and devices, which interact with cells and tissues at the molecular level with a high degree of specificity, are of immense requirement in the field of medicine to effectively combat infectious diseases, control cell proliferation and restore normal functioning of the human system. Nanoparticles of metals like gold, silver and platinum have myriad applications, particularly as optical contrast agents, multimodal sensors and in photothermal therapy^1, 2^. With the appreciation of the novel and/or enhanced properties of the metals at nanosizes, extensive research is being carried out in recent years for exploring the applications of nanoparticles in different fields like textile, chemical, pharmaceutical, medical and many others^3–6^. Silver nanoparticles are the most commonly used nanomaterials due to their unique electronic, optical, mechanical, chemical and biological properties which confer them a wide range of applications in different fields, especially in the sectors of health and environment^7–9^. There are several synthetic methods for the production of nanoparticles, and their characteristics like particle size, morphology, crystallinity, shape and properties could be engineered depending on the desired function. The medicinal properties of silver and its compounds are known from ancient times and hence are in use in household and jewellery items till date.

Chemical syntheses of nano-particles have disadvantages and therefore, researchers of late, are investigating the use of biological agents, specifically the plant extracts owing to their easy availability, cost effective and environmental friendliness. A careful examination of the related literature exposed the use of different types of plants (vegetable yielding as well as ornamental and/or avenue plants) for biological synthesis of silver nanoparticles. But green leafy vegetables, which are regularly consumed in the human diet in good amounts on a daily basis due to their nutritive value and palatability, have not been encountered in our survey. Hence, the present study aimed at synthesizing silver nanoparticles using the aqueous leaf extract of the green, leafy vegetable *Rumex acetosa* (sorrel) was attempted. The positive results of this study are expected to give impetus for the synthesis of nano-particles with the extracts of leafy vegetables of wide and daily use and thereby improve the therapeutic acceptability of the silver nano-particles with least or no toxicity. The organic capping material of the Ag NPs synthesized with the extracts of edible plants, mainly comprise of human compatible phytochemical constituents and hence have remote possibilities of eliciting immune reactions in the recipient subjects, with the exception of human individual-specific allergies. In addition, the beneficial phytochemical constituents would enhance the functional properties of the Ag NPs.

## Results and Discussion

### Synthesis of silver nanoparticles

Biological method of metal nanoparticle synthesis overrides the problems associated with physical and chemical methods and includes economical and eco-friendly aspects. Biosynthesis of nanoparticles are classified under ‘bottom up approach’ that mostly involves oxidation/reduction reactions catalyzed by the microbial enzymes or the plant phytochemicals^10^. In general, the 3 components viz. the solvent medium, the reducing agent and the stabilizing agent involved in the biomimetic synthesis of nanoparticles are non-toxic, eco-friendly and derived from living resources. Among the biological sources, plant extracts are easily available, safe and normally non-toxic and possess a variety of metabolites that mediate the reduction of silver ions. The major phytochemicals include terpenoids, flavones, ketones, aldehydes, amides and carboxylic acids. Water soluble components like flavones, organic acids and quinones cause immediate reduction of the ions. Several plant species whose extracts have been utilized in the preparation of silver nanoparticles are presented in various research reports^10, 11^. Three types of benzoquinones viz. cyperoquinone, dietchequinone and remirin were found to be associated with a direct reduction of Ag^+^ ions and formation of silver nanoparticles^12^. Phytochemical analysis of the aqueous leaf extracts of *R. acetosa* revealed the presence of tannins, saponins, flavonoids, terpenoids, carbohydrates and xanthoproteins. The synthesis and stability of the obtained Ag NPs are due to the action of these phytochemicals, especially the flavonoids and terpenoids^13, 14^.

### Characterization of Ag NPs

**Figure 1:**
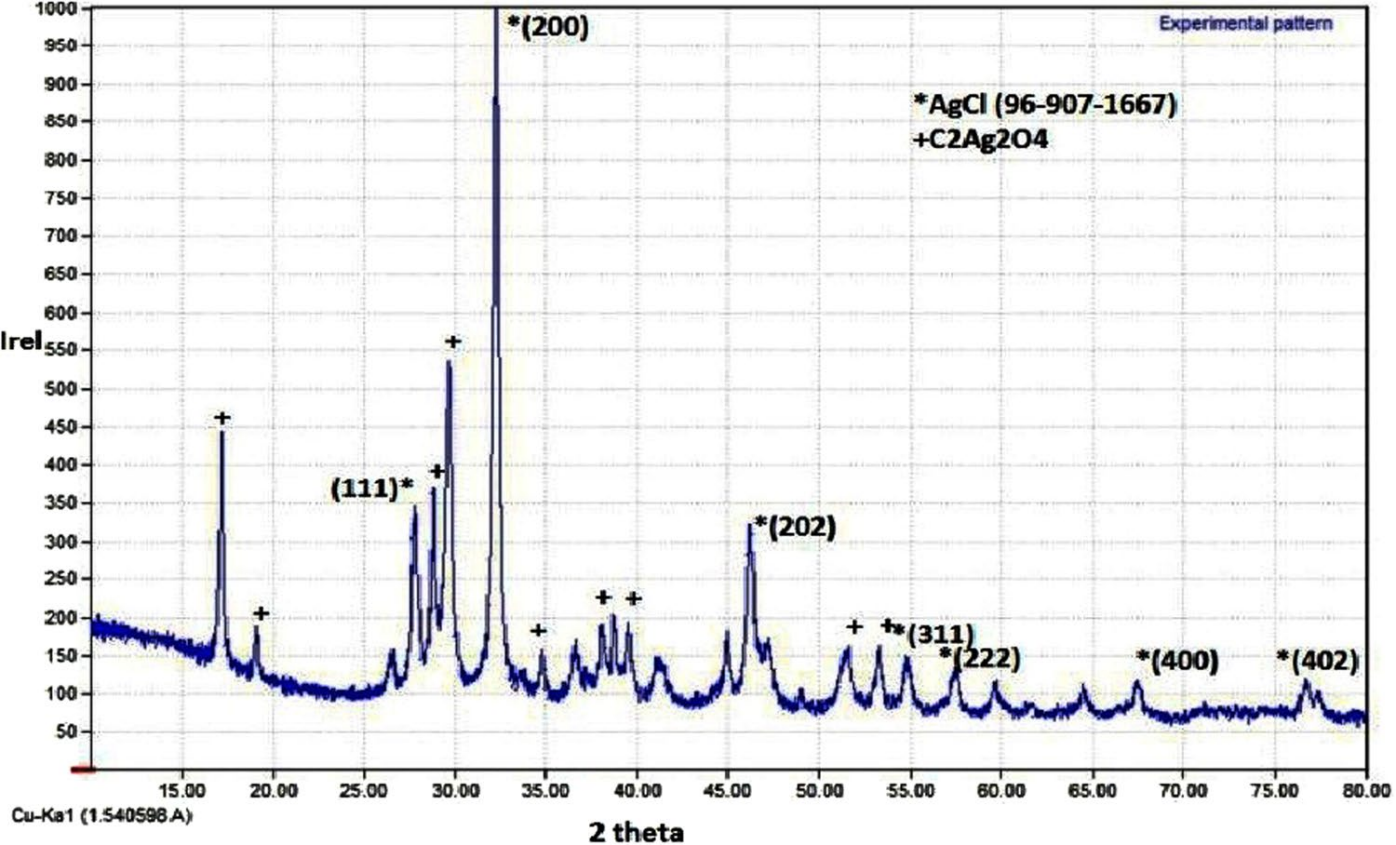
XRD pattern of the crystal structure of the silver nanoparticles obtained by green synthesis with the leaf extract of *R. acetosa*.

UV-vis spectral analysis indicates that the surface Plasmon spectra for the Ag NPs with brownish black color were centered approximately at 448 nm (Fig. S1). FT-IR spectral analysis (Fig. S2) demonstrated a peak at 652 cm^−1^ that indicates the C-H & N-H out of plane bending vibration. A sharp peak at 1637 cm^−1^ indicates the N-H bending vibration. The peaks at 3345 cm^1^, 3724 cm^−1^, 3770 cm^−1^, 3836 cm^−1^, 3886 cm^−1^, 3923 cm^−1^, and 3962 cm^−1^ indicate the involvement of N-H amines and O-H bonds (Table S1). Phase analysis of obtained XRD data using Match2 software indicated 17 peaks, out of which 6 peaks matched with AgCl (Chlorangyrite, 100%) corresponding to 2θ angles 27.82°, 32.26°, 46.21°, 54.8°, 57.48°, and 76.66°. The other major composition was silver oxalate (C_2_Ag_2_O_4_) with diffracting angles of 17.19°, 19.08°, 28.81°, 29.67°, 34.78°, 38.72°, 39.53°, 51.56°, and 53.25° (Fig. 1). Size and shape distribution of nanoparticles obtained by green synthesis using plant extracts are influenced by several factors like nature of the plant extract, pH of the solution, temperature of the reaction and others^11^. Uniform size and shape distribution of Ag NPs could be obtained by optimizing the above parameters for each type of plant extract under investigation.

**Figure 2:**
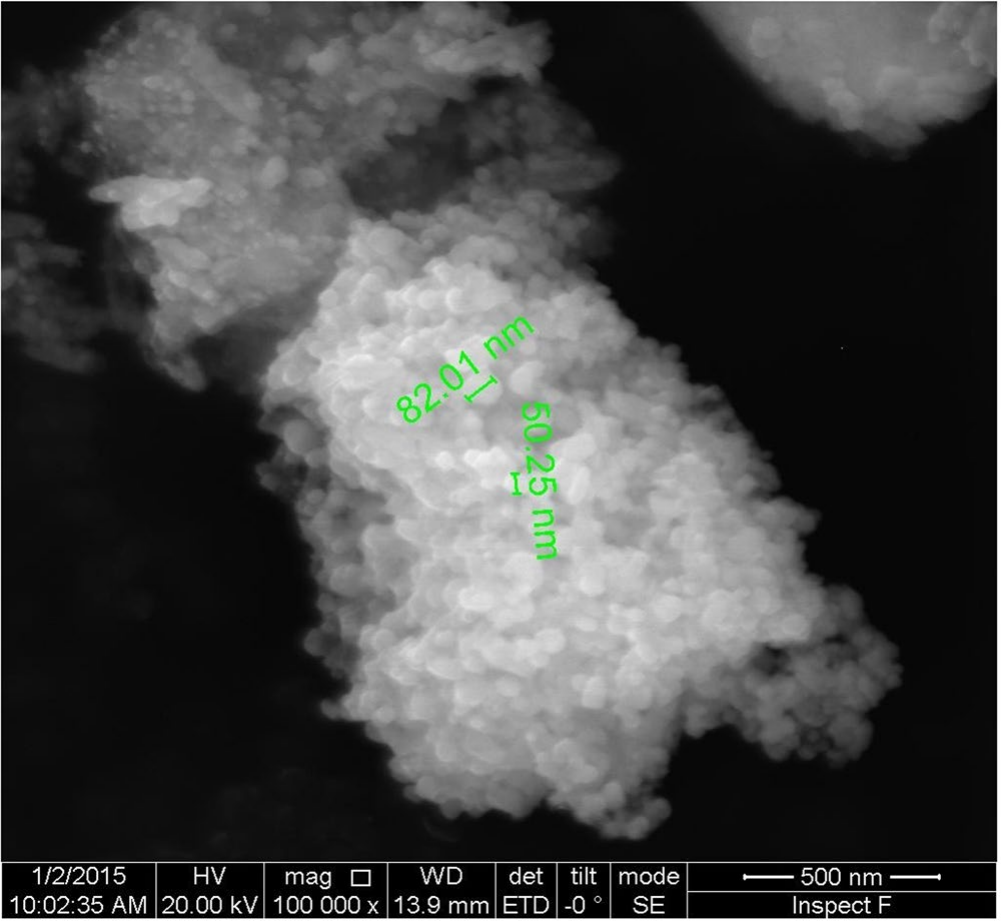
Micrograph of synthesized silver nanoparticles with the extract of *R. acetosa* obtained by Scanning Electron Microscopy.

**Figure 3:**
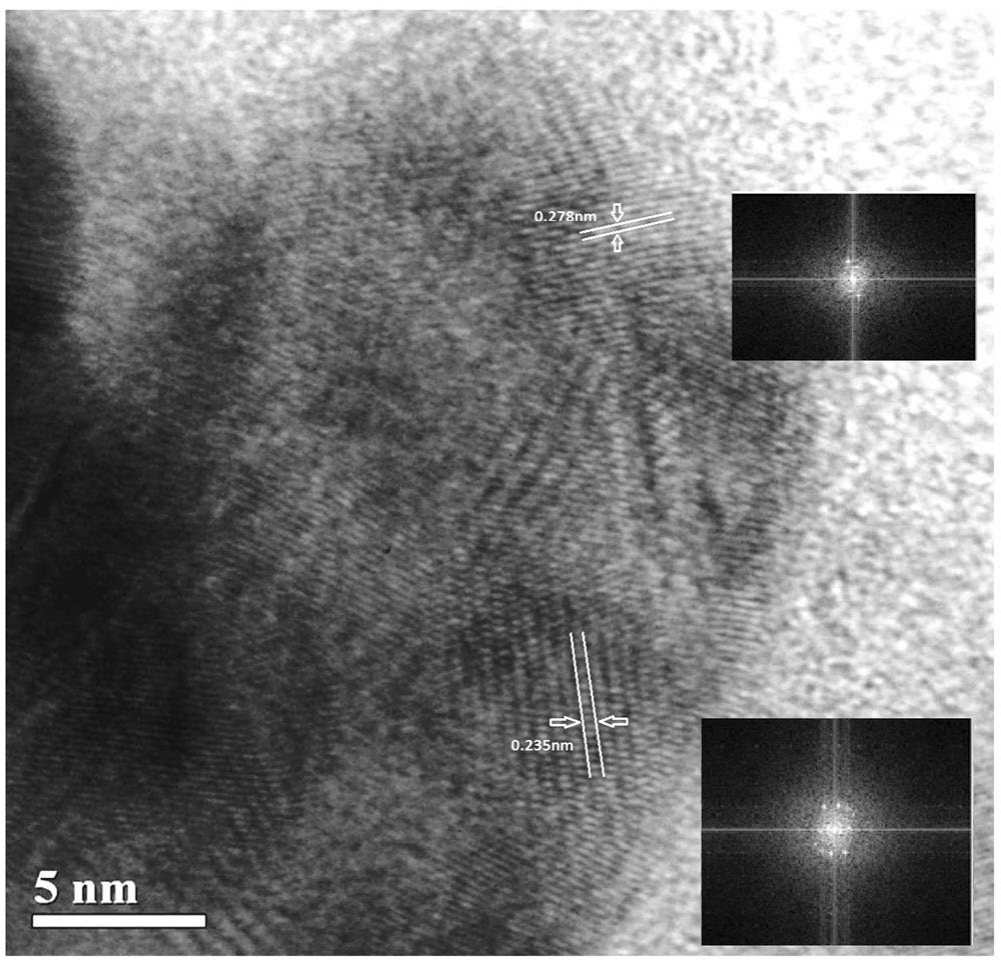
HRTEM images of synthesized silver nanoparticles.

**Figure 4:**
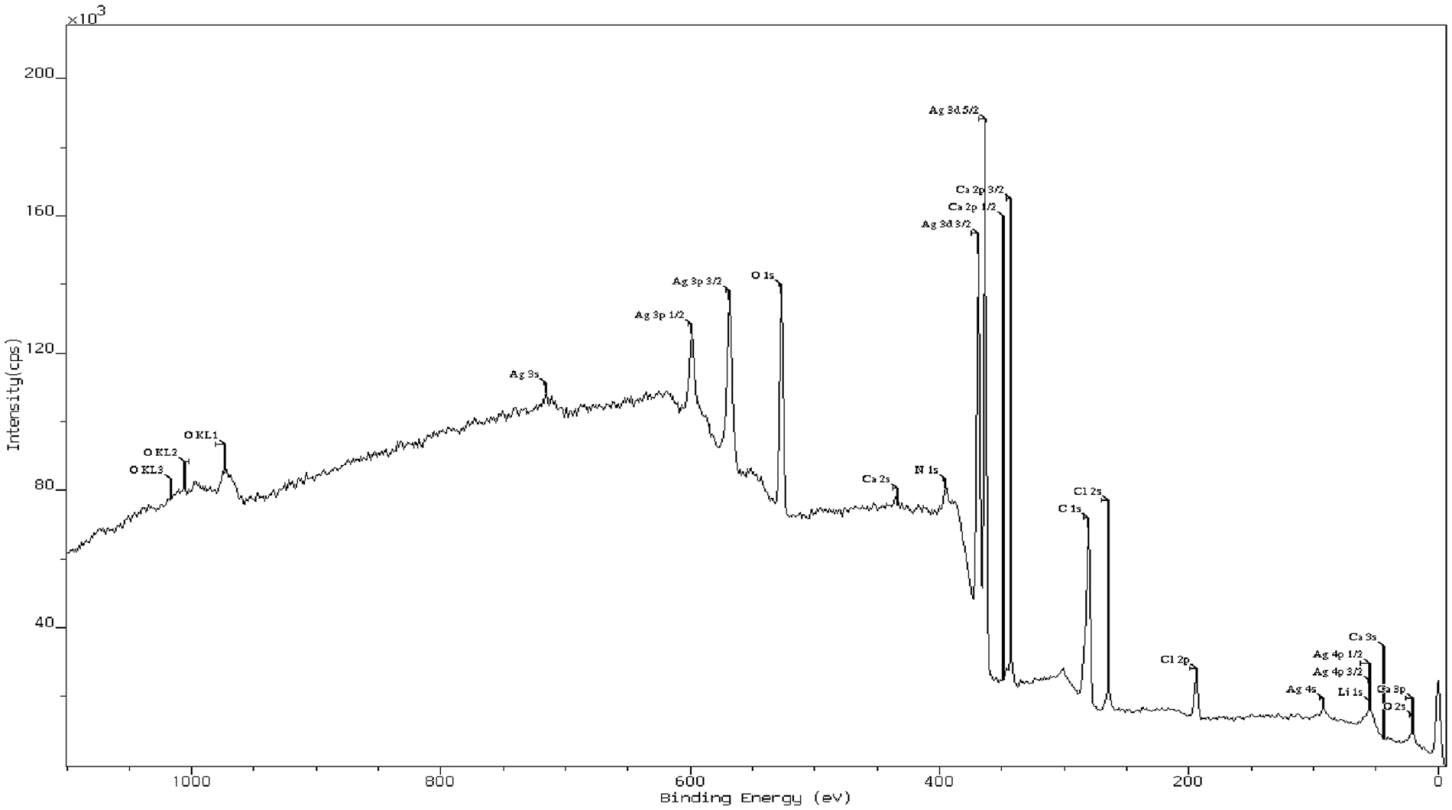
XPS survey spectrum for the synthesized Ag NPs showing the peaks for Ag, C, O, Cl and other minor constituents.

The synthesized silver nanoparticles were found to have a diameter of 35.34 nm, as calculated from Scherrer equation using the obtained XRD data (2θ = 32.31; FWHM = 0.2342; Relative intensity = 100%). SEM images indicate the particles to be predominantly spherical and with a size ranging between 50.25 nm and 82.01 nm (Fig. 2). Data obtained from TEM analysis suggest the particles to be ranging in size, predominantly between 5 and 40 nm (Fig. S3), the maximum number of particles in the size range between 15 and 20 nm (Fig. S4). A summary of these data suggests that the particles obtained by the synthesis with the leaf extract of *R. acetosa* are spherical and are largely in the size range between 15 nm and 50 nm, the maximum being about ∼80 nm. The calculated inter planar distances (Gaton digital micro graph) for the obtained HRTEM images (Fig. 3 and Fig. S5a,S5b) varied from 0.235 nm to 0.278 nm through 0.26 nm, suggesting the predominant occurrence of phase forms of Ag and AgCl. The XPS survey spectrum (Fig. 4) showed clearly the peaks for Ag, Cl, C and O along with insignificant presence of Ca, Li and N which are normally present in the leaf extracts. The core levels of Ag 3d, O1s and C1s peaks were fitted with Gaussian line shapes that gave reasonable agreement with the experimental results (Fig. S6a–S6c). The C1s signal, deconvoluted as two peaks by fitting, was attributed to the carbon atoms in aliphatic chain, C-C and carboxylic carbon, O-C=O with the binding energies of 284.8 eV and 287.5 eV respectively. The Ag 3d_5/2_, and Ag 3d_3/2_ with the binding energies of 368.36 eV and 374.40 eV respectively, with a splitting of approximately 6 eV signifies the formation of carboxylates protected Ag^0^ metal (monolayer protected clusters, MPC)^15, 16^. The core level O1S peak, appearing as a very broad peak with binding energy of 532.4 eV may be resolved to three peaks corresponding to C=O (carbonyl and carboxyl, 531.6 eV), C-O (hydroxyl, 532.4 eV) and O-H (hydroxyl, 533.35 eV)^17^.

**Figure 5:**
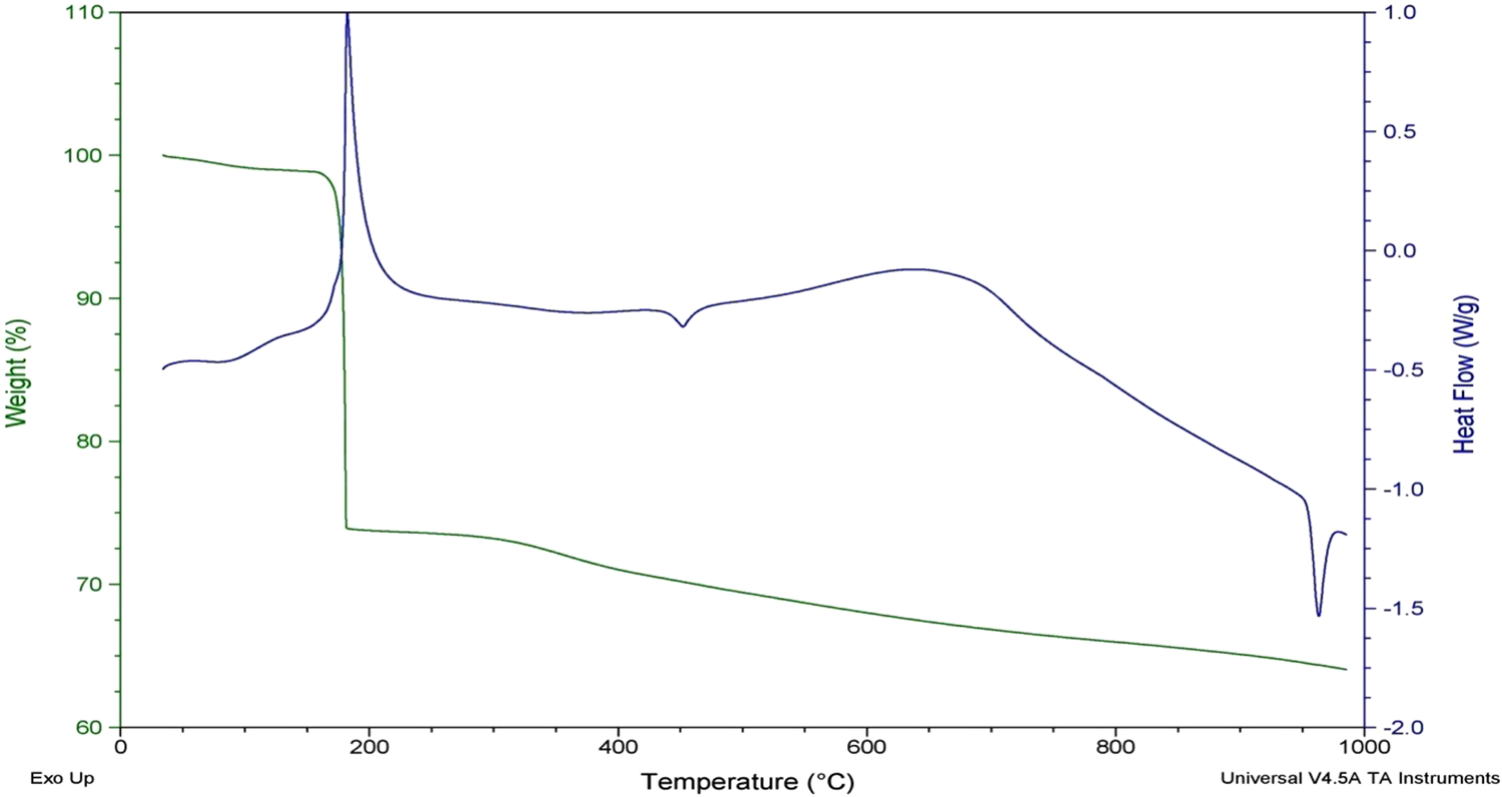
DSC-TGA of the synthesized silver nanoparticles with the leaf extract of *R. acetosa*.

The thermal decomposition of the sample occurred at 181 °C, accompanied with a weight loss of 37.75%, followed by a marginal decrease in the mass with further increase in temperature to 950 °C. At 181 °C, the organic capping compounds on the Ag NPs must have decomposed. From the decomposition temperature, the activation energy of desorption of organic matter was calculated by the Redhead analysis with the equation, E_d_ = RT_P_ (ln(ν_1_T_P_/β) –3.64), where ν_1_ is pre exponential factor, T_P_ is the decomposition temperature and β is the linear heating rate (assumed first order Kinematics) and was found to be 30.16 kcal/mole which is comparable to the earlier reports^15^. From the results of DSC, a sharp exothermic peak at 200 °C could be noted along with two small endothermic peaks, one at 455 °C and the other at 960 °C corresponding to the melting point of metallic silver (Fig. 5). From the energy dispersive X-ray analysis, the synthesized nanoparticles are found to be significantly constituted by Ag (64.55%) along with C, N, O and Cl corresponding to 19.2%, 2.56%, 7.23% and 6.46% respectively (Fig. S7).

### Antioxidant and Cytotoxic activities

Different types of free radicals, including oxygen radicals and organic hydroperoxides are reported to play a significant role in cytotoxicity and carcinogenesis^18, 19^. Many tumor promoters induce a ‘pro-oxidant state’ in their target tissue resulting in carcinogenic activity. Natural (vitamins C and E) and synthetic (ethoxyquin, butylated hydroxyanisole, butylated hydroxytoluene etc.) antioxidants are shown to inhibit carcinogenic chemicals by modifying their activation, detoxification and mutagenicity, in addition to their ability of scavenging reactive carcinogen metabolites and free radicals^20^. In the present study, an attempt is made to examine the anti-oxidant effects of biologically synthesized Ag NPs *in vitro* and their influence on the metabolic activity of human osteosarcoma cell lines. Biologically synthesized Ag NPs, with the aqueous leaf extract of *R. acetosa*, were found to scavenge H_2_O_2_ in a concentration dependent manner, the highest percentage scavenging activity of 99.02% attained at 200 μg/ml which is higher than the percentage scavenging obtained with 200 μg/ml of the standard ascorbic acid. Similarly, considerable antioxidant activity was demonstrated by the Ag NPs with respect to DPPH and NO inhibition. However, the DPPH scavenging activity was significantly lower than the standard, while NO inhibition by the Ag NPs was better and closer to the values obtained for ascorbic acid. The results obtained for different *in vitro* antioxidant assays are plotted in Figures S8–S11 with their corresponding linear regression equations. IC75 for Ag NPs and the standard ascorbic acid for different antioxidant tests were calculated from the regression equations and are presented in  μg/ml in Table S2. The statistical results presented (Tables S2 and S3) suggest that the synthesized Ag NPs, although have a low antioxidant potential in comparison with the standard ascorbic acid, are still a good choice as anti-oxidant therapeutics.

**Figure 6:**
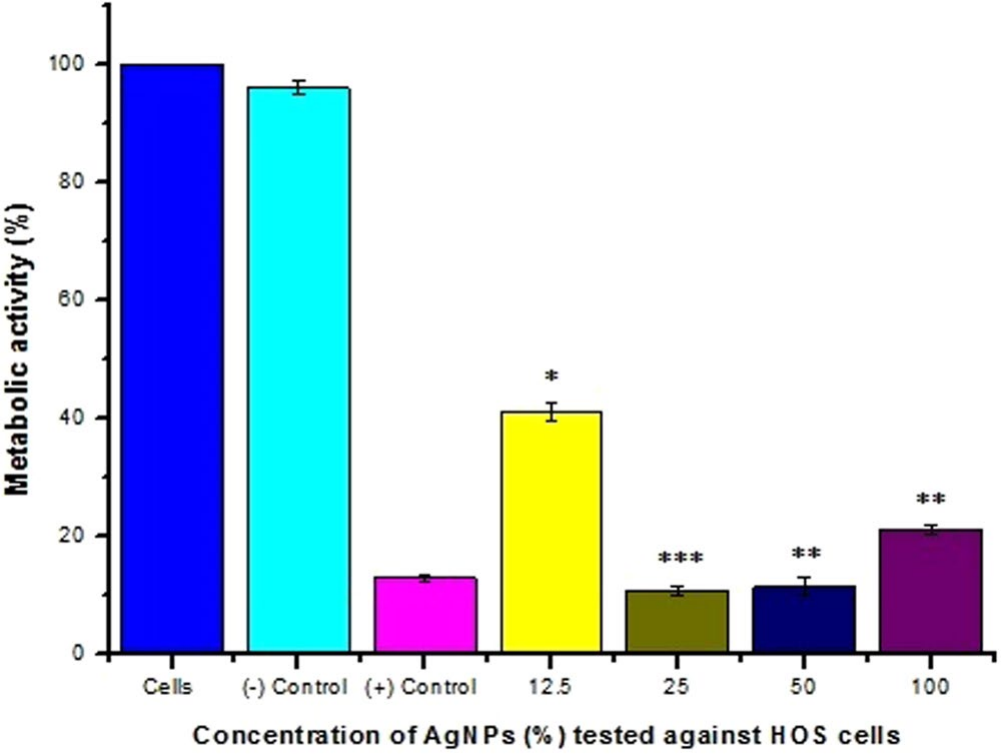
Evaluation of Cytotoxic activity of different concentrations (%) of Ag NPs against human osteosarcoma (HOS) cell lines. Negative control: High density polyethylene; Positive control: 1.3 mg/ml of phenol in culture medium.

MTT assay is based on the number of mitochondria of cells expressed as percent of metabolic activity. The results of the assay evaluated after 24 hours of incubation (Fig. 6) showed that the highest significant (p < 0.05; ***) decrease in the metabolic activity to 10.81% was obtained at 25% extract concentration as compared to the decrease in residual metabolic activity to 41.17% corresponding to 58.83% inhibition at 12.5% extract concentration (p < 0.05; **), all with reference to untreated cells. However, the metabolic activity of the cells gradually increased to 11.49% at 50% extract concentration (88.5% inhibition; **) and finally reached 21.09% at 100% concentration (**) of the extract (78.91% inhibition). The marginal revival in metabolic activity at 50% and 100% extract concentrations was unexpected, yet interesting and the reasons to be investigated. The obtained results, analyzed statistically with one way ANOVA (Table S3), suggest that 25% concentration of the Ag NPs has a significant inhibitory effect on the activity of human osteosarcoma cell lines. Also, the cells did not get further inhibited with the increase in the concentrations of the extract (50% and 100%), probably because at higher concentrations, aggregates of the Ag NPs could have been formed and therefore resulted in decreased concentration of active Ag that could penetrate the cell and impair the mitochondrial membrane potential, structure and/or mitochondrial enzymes^21, 22^. Similar studies on the effect of chemically synthesized Ag NPs of 10 ± 1.0 nm on CHO (Chinese Hamster Ovary) cell lines demonstrated a clear dose dependent inhibition of cell viability (which is in sharp contrast to our results in higher concentrations) probably due to smaller particle size as compared to the Ag NPs of approximately 35 nm used in the present study^23^. Further insight into the results prompts the idea that the toxicity of the Ag NPs could be moderated through appropriate selection of particle size, depending on the extent of inhibition required.

In general, nanoparticles are taken up by mammalian cells by one or more models of the three endocytotic transport mechanisms viz. pinocytosis, phagocytosis and receptor-mediated endocytosis. Studies on the interaction of silver nanoparticles with mitochondria confirmed that the Ag NPs are endocytotically taken up by cells and are localized internally outside the nucleus, endoplasmic reticulum, golgi and mitochondria. They were also reported to form agglomerates in the perinuclear zone, very close to the mitochondria^24^. In this study of Bressan *et al*., it has been demonstrated that the mitochondrial functionality reduced in the presence of Ag NPs, although the nuclei were undamaged and there were no signs of apoptosis^24^. Corroborating with these findings, we could notice the increase in metabolic activity of the Ag NP treated cells at higher concentrations, which is indicative of ‘switch over’ of the metabolisms of cells from ‘aerobic’ to ‘anaerobic’ rather than immediate apoptosis. Nanoparticle accumulation around the mitochondria might disrupt the respiratory chain and generate reactive oxygen species, but these species get inhibited by virtue of the antioxidant activity of Ag NPs. Thus the Ag NPs could ideally serve as therapeutic candidates, provided their chosen concentrations are non-toxic, for preventing cellular damage through the inhibition of reactive oxygen species (ROS) that are produced either by metabolic errors and/or damage to cellular organelles^19^.

### Antibacterial activity

Antibacterial action of silver nanoparticles is ascribed to one or more of their abilities to anchor to the cell wall, cause pits and alter permeability of the cell membrane resulting in death of the cells^25^, generation of free radicals that induce porosity in the cell membrane, inactivation of enzymes by blockage of thiol groups, inhibition of respiratory enzymes by silver ions causing the release of reactive oxygen species (ROS), soft acidic action of Ag with soft basic action of sulphur and phosphorus resulting in destruction of DNA^26^, and/or inhibition of bacterial growth through the inhibition of signal transduction by dephosphorylation of peptide substrates on tyrosine residues^27^. Physico-chemical characteristics viz. size, pH, ionic strength and capping agents are the major factors influencing their antimicrobial property^28–31^. Although the exact mechanism(s) underlying the property is under intense scrutiny, silver in its ionic state (Ag^+^) is reported to form complexes with nucleic acids through it’s preferential interaction with nucleosides^32^. The positively charged silver ions released from Ag NPs^30^ are suggested to get attracted towards negatively charged bacterial cells^29^, accumulate inside the membrane and subsequently penetrate into the cells causing damage to cell wall and/or cell membranes^25^.

**Figure 7:**
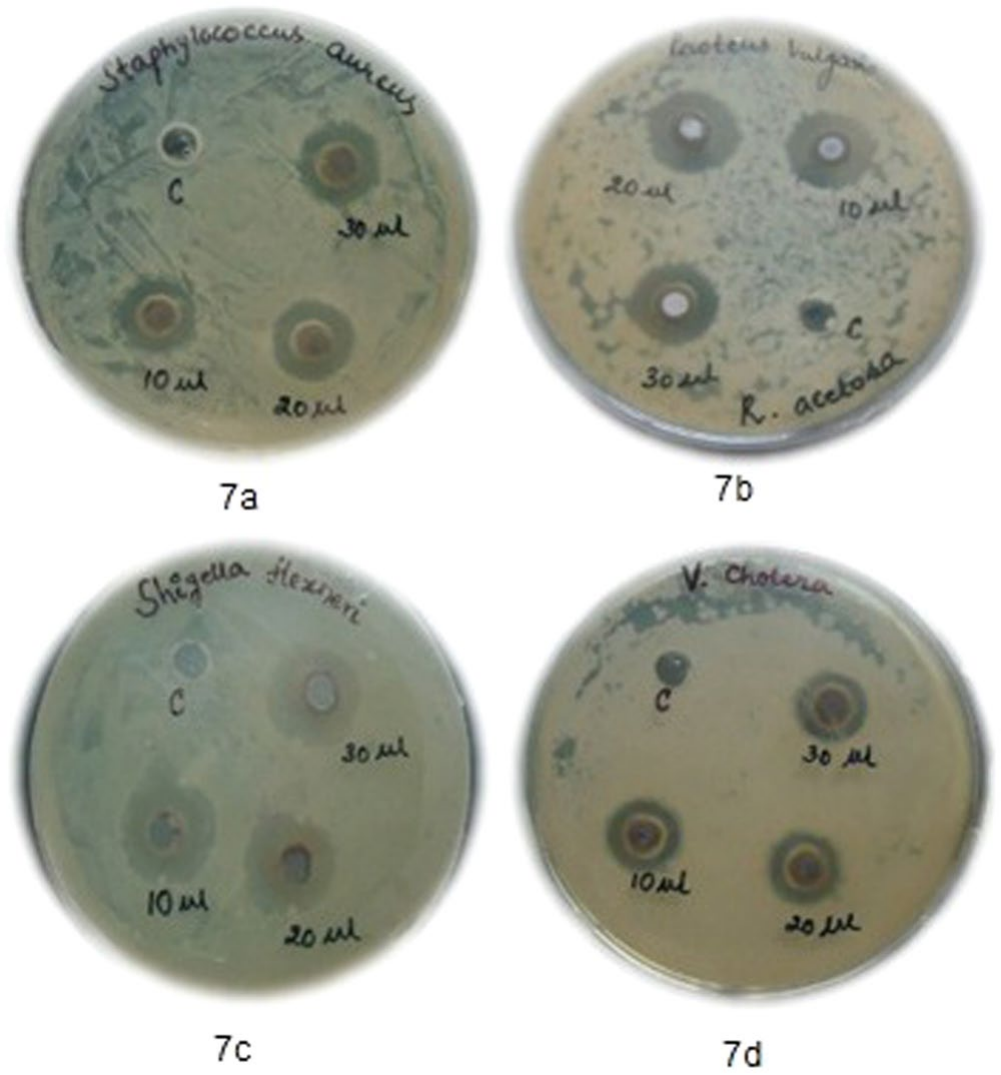
(**a to d**) Zones of Inhibition obtained with the silver nanoparticles synthesized using the leaf extract of *R. acetosa*.

Both gram positive and gram negative bacterial strains present major public health problems which are further complicated by the emergence of multi-drug resistant strains^33^. Gram negative bacteria are more susceptible than the gram positive because of their thinner cell walls (less amounts of negatively charged peptidoglycans) and therefore with less number of negative charges to lock up Ag^+^ ions. The increasing ease to prepare nanoparticles of desired size and shape that are critical in conferring their properties is definite to revolutionize their therapeutic applications and provide a handle to develop effective control methods for infections. Antimicrobial activity of nanoparticles has majorly been studied with pathogenic bacterial strains like *E. coli* and *S. aureus* ^34–37^. From the measured zones of inhibition obtained with the sixteen clinical isolates (Table 1), it is clear that the Ag NPs are effective against both gram positive and gram negative species and the zones varied between ~12 mm and ~18.5 mm. Of the four gram positive species tested, *Staphylococcus sps*. are better inhibited than *B. cereus and Enterococcus sps*. Among the gram negative, *E. coli*, *V. cholera* and *Salmonella sps*. are strongly inhibited at the lowest tested dose of 500 μg/well (Fig. 7a to 7d). Although, a comparison of the present results with the antibacterial potential of chemically synthesized Ag NPs against *Staphylococcus sps*. (Pathogenic gram positive), *Salmonella typhi* (pathogenic gram negative) and *Pseudomonas sps*. (gram negative)^38^ suggests the lower antibacterial potential (requirement of a higher dose) of biologically synthesized silver nanoparticles, yet the silver nanoparticles synthesized with biological materials are preferred choice for their least/no toxicity to host cells.

**Table 1:** Zones of inhibition (mm) obtained with Ag NPs synthesized using the extract of *R. acetosa* against human clinical isolates.

S. No.	Name of the organism	Gram’s reaction and cell morphology	Zone of Inhibition (mean ± S.D.) in mm obtained with 50 μg/μl concentration of aqueous suspension of Ag NPs		
			10 μl	20 μl	30 μl
1	*Bacillus cereus*	Gram positive, rod	15.5 ± 0.5	16 ± 0	16 ± 0
2	*Enterococcus sps*.	Gram positive, cocci	15.3 ± 1.25	15.3 ± 1.25	15.3 ± 1.25
3	*Escherichia coli*	Gram negative, rod	16.5 ± 0.5	17 ± 0	17.5 ± 0.5
4	*Klebsiella sps*.	Gram negative, rod	14.5 ± 0.5	15 ± 0	15 ± 0
5	*Proteus mirabilis*	Gram negative, rod	14 ± 0	15 ± 0	14.5 ± 0.5
6	*Proteus vulgaris*	Gram negative, rod	15 ± 0	15 ± 0	15.5 ± 0.5
7	*Pseudomonas sps*.	Gram negative, rod	11.7 ± 0.5	12.7 ± 0.5	13 ± 0.8
8	*Salmonella paratyphi A*	Gram negative, rod	12.2 ± 1.5	12.4 ± 2.15	12.5 ± 1.7
9	*Salmonella typhi*	Gram negative, rod	14.3 ± 0.5	14.3 ± 0.5	14.3 ± 0.5
10	*Salmonella typhimurium*	Gram negative, rod	15 ± 0	15.5 ± 0.5	15 ± 0
11	*Serratia*	Gram negative, rod	16 ± 0	16.3 ± 0.5	16.7 ± 0.5
12	*Shigella dysentery A*	Gram negative, rod	16.5 ± 0.5	17 ± 0	18.5 ± 0.5
13	*Shigella flexneri*	Gram negative, rod	16 ± 0	17.3 ± 1.25	17.3 ± 2
14	*Staphylococcus aureus*	Gram positive, cocci	18 ± 0	18 ± 0	18.5 ± 0.5
15	*Staphylococcus epidermidis*	Gram positive, cocci	17 ± 0	18 ± 0	18.3 ± 0.5
16	*Vibrio cholera*	Gram negative, comma shaped	15.5 ± 0.5	15.5 ± 0.5	15.5 ± 0.5

## Conclusions

Silver is known from ancient times for its antimicrobial activity and its efficacy is found to increase manifold at nano-level due to unique physical and chemical properties. Presently, biological synthesis of Ag NPs has become the preferred choice, more particularly with the plant extracts for their wide/easy availability, non-toxicity/environmental friendliness and cost effectiveness. In this study, the Ag/AgCl NPs synthesized using the leaf extracts of the edible green leafy vegetable of *R. acetosa* have demonstrated high antimicrobial, antioxidant, and cytotoxicity against the tested human osteosarcoma (HOS) cell lines. Their valuable potential in medical applications gives them an edge as compared to other materials and the reported toxicity in higher concentrations could be addressed by using the edible plant leaves like those of *R. acetosa* with suitable phytochemical constituents for the formation of Ag NPs. Also, suitable antidotes to prevent toxicity to healthy cells should be explored to make better use of these particles for a safer environment and public health.

## Methods

All the chemicals used in the study were of analytical grade, purchased from Himedia (Mumbai, India) and Merck (New Delhi, India). The experiments were conducted in triplicate and the mean values with standard deviation are reported. Student’s ‘T’ test, regression analysis and analysis of variance (ANOVA) were performed with SigmaPlot 13.0 trial version.

### Preparation of leaf extract and synthesis of AgNPs

Healthy twigs of *R. acetosa* (sorrel), purchased from the local vegetable market, Guntur, Andhra Pradesh, India, were thoroughly washed and air dried. Fine chopped 50 g leaves were boiled in 250 ml of deionized water for 15 min, filtered first through muslin cloth followed by Whatman No. 1 filter paper. Phytochemical analysis of the filtered leaf extract was done following standard procedures^39^. The filtered extract (pH 7.5) was used within 3 hours for the preparation of Ag NPs. Leaf extract was added to 6 mM AgNO_3_ solution in 1:10 ml ratio and left for about 30 to 60 min to change color to brown. Thereafter, a high speed spin 10,000 rpm for 20 min. discarded the supernatant and kept the pellet/sediment for drying at 60 °C in hot air oven for about 12 hours. The dried pellet was powdered, autoclaved and stored at 4 °C.

### Characterization of Ag NPs

The Ag NPs were characterized by spectra obtained by UV-vis spectrophotometer (Shimadzu) at 380 to 500 nm. Fourier transform infrared (FT-IR) spectra (Bruker, UK) were obtained in the range of 400 to 4000 cm^−1^ by KBr pellet method to determine the functional groups. The metallic Ag NPs were examined using an x-ray diffractometer (PANlytical Xpert Pro) equipped with a CuKá radiation source at a generator setting of 40 kV/30 mA and data was collected in the range of 9.971°–99.955°. For SEM-EDAX, a small quantity of the powder was spread over the carbon tape and inserted into the chamber. A series of images were taken (System Quanta Inspect F) at different voltages and magnifications, to understand the morphology and chemical composition of the sample. Transmission electron microscopic examination of the particles was undertaken to determine the size distribution of Ag NPs. JEOL_JEM-2010 instrument was used to obtain the high resolution transmission electron microscopy (HRTEM) images of the samples and the selected area electron diffraction (SAED). For these experiments, the samples were subjected to ultra-sonication in ethanol medium to disperse the fine powder onto the copper grids. These TEM images were used to investigate the morphology while the SAED confirmed the nature of crystallinity. XPS experiments were carried out on the Krotas Axis Ultra^DLD^ Model at the base pressure of 1 × 10^−9^ Torr and the working pressure at 5 × 10^−9^ Torr, with Mono Al Kα as irradiation source of energy (1486.71 eV operated at 15 KV and 5 mA) to determine the chemical composition and the valence states of the prepared material. Thermo-gravimetric-differential scanning calorimetry (TGA-DSC) analysis with 50 mg of the powder in a small alumina crucible (Universal V4.5 A TA instruments of model SDT 2600 V20.9 Build 20) was carried out using N_2_ gas with a ramp (heating) of 20 °C/min.

### Antioxidant and Cytotoxic activities

#### DPPH radical scavenging activity

The DPPH assay as described by Hsu *et al*. was carried out with a few modifications^40^. The reaction was prepared by mixing 2.5 ml of deionized water, 1.5 ml of 0.3 mM DPPH in methanol and 1.0 ml of varying concentrations of Ag NPs (40, 80, 120, 160, and 200 μg/ml), vortexed and incubated at room temperature for 30 min in the dark. For each reaction tube, a blank containing the same constituents except DPPH was set up and the absorbance of the blank was subtracted from the absorbance of the experimental tubes as a correction factor. The reduction of DPPH free radical was measured by reading the absorbance at 517 nm using UV-vis spectrophotometer. A control reaction containing 2.5 ml of deionized water and 1.5 ml of 0.3 mM DPPH was set up simultaneously under the same conditions as the experimental tubes. Ascorbic acid was used as positive control and the percentage inhibition of DPPH free radical was determined from the measured absorbance values,

1 $${\rm{DPPH}}\,{\rm{free}}\,{\rm{radical}}\,{\rm{scavenging}}\,{\rm{activity}}\,( \% )=({{\rm{A}}}_{{\rm{c}}}-{{\rm{A}}}_{{\rm{t}}})/{{\rm{A}}}_{{\rm{c}}}\times 100$$

where A_c_ is the absorbance of the control and A_t_ is the absorbance of the test/standard. The IC_75_ value was calculated from the graph plotted with the percentage scavenging activity against the Ag NP concentration.

#### Hydrogen peroxide scavenging activity

Scavenging of H_2_O_2_ by the Ag NPs was estimated by the method given by Ruch *et al*. with certain modifications^41^. First, 40 mM solution of H_2_O_2_ in 0.1 M phosphate buffer (pH 7.4) and 0.6 ml of it was then added to test tubes containing 3.4 ml of phosphate buffer with varying concentrations of Ag NPs (40, 80, 120, 160, and 200 μg/ml) suspended in 1:1 methanol and water, and incubated for 10 min. Similar concentrations of ascorbic acid in place of silver nanoparticles were run as standard. The absorbance of the solution was measured at 230 nm against a blank solution of phosphate buffer and the extract containing varying concentrations of Ag NPs, without H_2_O_2_. Absorbance of 40 mM hydrogen peroxide was used as a control. The percentage of H_2_O_2_ radical scavenging activity was calculated as:

2 $${{\rm{H}}}_{{\rm{2}}}{{\rm{O}}}_{{\rm{2}}}\,{\rm{radical}}\,{\rm{scavenging}}\,{\rm{activity}}\,( \% )=({{\rm{A}}}_{{\rm{c}}}-{{\rm{A}}}_{{\rm{t}}})/\mathrm{Ac}\times 100$$

where A_c_ is the absorbance of the control and A_t_ is the absorbance of the test/standard. The IC_75_ value was calculated from the graph plotted with the percentage scavenging activity against the Ag NP concentration.

#### Reducing power assay

Reducing power of varying concentrations of Ag NPs (40, 80, 120, 160, and 200 μg/ml) suspended in 1:1 methanol and water were determined by the method of Oyaizu *et al*.^42^. Different reaction mixtures each containing 1.0 ml of the chosen concentration of the sample, 2.5 ml of 0.2 M phosphate buffer (pH 6.6) and 2.5 ml of 1% potassium ferricyanide were prepared and incubated at 50 °C for 20 min in a hot water bath. Then, 2.5 ml of 10% trichloroacetic acid was added to the reaction mixture and was centrifuged at 3000 rpm for 10 min. Finally, 2.5 ml of the upper layer of the solution was mixed with 2.5 ml of distilled water and 0.5 ml of ferric chloride. Absorbance of the resulting solution was measured at 700 nm against a blank containing all the reagents except the sample. A stronger absorbance is indicative of higher reducing power.

#### Nitric oxide scavenging activity

Biologically, nitric oxide free radicals (NO.) are generated by the action of specific nitric oxide synthases that metabolize amino acids like arginine. *In-vitro*, sodium nitroprusside decomposes in aqueous solution at a physiological pH of 7.2 and releases NO free radicals that react with oxygen to produce stable nitrites and nitrates, which in turn, can be determined by Griess reagent^43^. Here, 2 ml of 10 mM sodium nitroprusside dissolved in 0.5 ml phosphate buffer saline (pH 7.4) was mixed with 0.5 ml of various concentrations of Ag NPs (40, 80, 120, 160, and 200 μg/ml) suspended in 1:1 methanol and water. The mixture was then incubated at 25 ± 2 °C for 150 min and absorbance was measured at 546 nm for all the test samples that served as control. Then, 2 ml of the incubated solution was added to 2 ml of freshly prepared Griess reagent (equal quantities of 2% sulphanilamide in 1.47 M HCl and 0.1% N-(1-naphthyl) ethylenediamine dihydrochloride in deionized water) and further incubated for 30 min. at room temperature. Absorbance at 546 nm was again measured after incubation with Griess reagent and NO inhibition was calculated as:

3 $$ \% \,{\rm{Inhibition}}\,{\rm{of}}\,{\rm{NO}}\,{\rm{radical}}={(A}_{{\rm{0}}}-{{\rm{A}}}_{{\rm{t}}})/{{\rm{A}}}_{{\rm{0}}}\times 100$$

where A_0_ is the absorbance before reaction and A_t_ is the absorbance after reaction with Griess reagent.

The results obtained were subjected to student’s ‘T’ test (to determine whether there is significant level of difference (‘*p*’ value) between the standard and the test samples) and regression analysis as well. From the regression equation, inhibitory concentrations were calculated.

#### Cytotoxic activity

For understanding cytotoxicity, MTT (methyl-thiazolyl-tetrazolium) assay (a colorimetric, indirect method for assessing mitochondrial activity as a function of cell growth and proliferation) was done^44^. The test is based on the reduction of yellowish aqueous solution of tetrazolium salt 3-(4,5-Dimethylthiazol-2–yl)-2,5-diphenyltetrazolium bromide, by dehydrogenases or other reducing agents present in metabolically active cells, to violet blue/purple colored water insoluble dye compound, formazan. Extract was prepared by incubating 100 mg of synthesized Ag NPs in 1 ml minimal essential medium supplemented with Fetal Bovine Serum at 37 ±1 °C for 24 ± 2 hours. After 24 hours, the extract was diluted with culture medium to get 50%, 25% and 12.5% concentrations. Cells cultured in normal medium were considered as cell control. Equal volume (100 μl) of various dilutions of test samples, extracts of negative control (high density polyethylene), cell control and positive control (13 mg/ml of phenol stock diluted to 1.3 mg/ml with the above culture medium) were placed on subconfluent layer (10^4^ cells/well) of Human Osteosarcoma (HOS) cells. After incubation of cells with various concentrations of test sample and controls at 37 ± 1 °C for 24 ± 2 hours, extracts and control medium were replaced with 50 μl MTT solution (1 mg/ml in medium without supplements), wrapped with aluminum foil and were incubated at 37 ± 2 °C for 2 hours. MTT solution was discarded and 100 μl of isopropanol was added to all wells and swayed the plates. The color developed was quantified by measuring absorbance at 570 nm using a spectrophotometer (Biotek Power wave XS). The data obtained for test samples was compared with cell controls.

### Anti-Bacterial activity

The antibacterial activity of the synthesized silver nanoparticles was evaluated against 16 human clinical isolates procured from the culture collection maintained in the department of microbiology of a local multi-specialty hospital. The clinical isolates were cultured in a nutrient agar medium (Hi-Media) containing yeast extract 3.0 g, peptone 5.0 g, NaCl 5.0 g, Agar 15.0 g, and distilled water 1 L (pH 7.2), under aerobic conditions at 37 °C for 24 hours and were handled strictly in accordance with the guidelines of Institutional Ethical Committee (IEC). Antibacterial activity was assessed with the cultured cells after 2 passes using the agar well diffusion method. Nutrient agar of 15 ml was dispensed in sterile conical flasks, inoculated with 0.2 ml of bacterial culture suspension, mixed gently, poured into sterile petri dishes and allowed to solidify. A sterile cork borer was used to punch wells and the base of each well was sealed with nutrient agar. Then 10, 20 and 30 μl of Ag NPs suspended in 5% DMSO (50 mg/ml) were added to the wells, while 5% DMSO was used as a negative control. The plates were incubated at 37 °C for 24 hours and zones of inhibition were measured by the Hi-Media scale. Antibacterial tests were done in triplicate and the average values with ± S.D. are reported in Table 1.

### Data availability statement

All the experimental methods included in our study are standard procedures and the references are provided.

### Guidelines for handling human pathogenic bacteria

The human pathogenic isolates procured from the department of microbiology of NRI Medical College, Guntur were strictly handled in accordance with the guidelines, and the experimental protocols laid down by the Institutional Ethical Committee were diligently followed. Human subjects were never involved in the study.

## Electronic supplementary material

Supplementary Information
