# Supplementary material for: Evaluation of therapeutic potential of the silver/silver chloride nanoparticles synthesized with the aqueous leaf extract of Rumex acetosa
Source: Sci Rep. 2017 Sep 14;7:11566. doi: 10.1038/s41598-017-11853-2 (PMC5599524; doi:10.1038/s41598-017-11853-2)

**Evaluation of therapeutic potential of the silver/silver chloride nanoparticles synthesized with the aqueous leaf extract of *Rumex acetosa***

K. Sobha^1*^, D. Pradeep^1^, A. Ratna Kumari^2^, Mahendra Kumar Verma^3^, K. Surendranath^1#^

^1^Department of Biotechnology, R.V.R. & J.C. College of Engineering (A), Guntur 522 019, Andhra Pradesh, India

^2^Centre for Biotechnology, Acharya Nagarjuna University, Nagarjuna Nagar, 522 010, Andhra Pradesh, India

^3^Department of Biological Sciences, Indian Institute of Science Education and Research, Bhopal-462 066, Madhya Pradesh, India

^1#^Department of Physics, R.V.R. & J.C. College of Engineering (A), Guntur 522 019, Andhra Pradesh, India

^*^Corresponding author, presently affiliated to the Department of Mathematics & Humanities

Email: [sobhakota2005@gmail.com](mailto:sobhakota2005@gmail.com)

Fax: 0863-2288274, 2350343

Mobile: 9985640105

**Supplementary material**

**Table S1** FTIR spectral characteristics of the silver nanoparticles synthesized with the leaf

extract of *R. acetosa*

| **S. No.** | **Absorption Peaks (cm^-1^)** | **Assigned functional groups** |
| --- | --- | --- |
| 1 | 652 | C-H & N-H out of plane bend; C-X (Halide) strong stretch |
| 2 | 1637 | N-H bend, C=O stretch amide, C=C stretch (alkenes) |
| 3 | 3345, 3724, 3770, 3836, 3886, 3923, 3962 | C-H, N-H (amines), O-H bond stretch |

**Table S2** Calculated IC_75_ values and Student’s ‘T’ test values for the synthesized *R. acetosa*

derived Ag NPs in comparison with the standard ascorbic acid.

| **S. No.** | **Antioxidant Assay** | **Regression equation** | **IC_75_ Concentration (μg/ml)** | | **Student’s ‘T’ test** | | **Inference** |
| --- | --- | --- | --- | --- | --- | --- | --- |
|  |  |  | Ag NPs | Ascorbic acid | T value | *p* value |  |
| 1 | DPPH | 0.138x+43.92; r^2^ = 0.990 | 225.2 | 49.8 | 4.2157 | 0.0029 | Significant |
|  | Ascorbic acid | 0.163x+66.88; ; r^2^ = 0.942 |  |  |  |  |  |
| 2 | H_2_O_2_ | 0.302x+32.85; r^2^ = 0.943 | 139.56 | 129.87 | 0.1784 | 0.8628 | Not significant |
|  | Ascorbic acid | 0.245x+43.18; ; r^2^ = 0.963 |  |  |  |  |  |
| 3 | NO | 0.121x+52.83; ; r^2^ = 0.982 | 183.2 | 89.94 | 2.1020 | 0.0687 | Not significant |
|  | Ascorbic acid | 0.172x+59.53; ; r^2^ = 0.943 |  |  |  |  |  |
| 4 | RP | 0.001x+0.089; r^2^ = 0.958 | - | - | 1.7811 | 0.1127 | Not significant |
|  | Ascorbic acid | 0.002x+0.076; r^2^ = 0.959 |  |  |  |  |  |

**Table S3** Results of One way Analysis of Variance (ANOVA) for the different antioxidant and

cytotoxicity tests performed with the Ag NPs derived with *R. acetosa*.

| **Test** | **Parameter** | **Sum of Squares (SS)** | **Degrees of Freedom (df)** | **Mean Square (MS)** | **F Value** | **P Value** | **Inference** |
| --- | --- | --- | --- | --- | --- | --- | --- |
| DPPH assay | Between groups | 1,697.288 | 1 | 1,697.288 | 22.212 | **0.002** | Significantly low |
|  | Within group | 611.300 | 8 | 76.413 |  |  |  |
|  | Total | 2,308.588 | 9 | - |  |  |  |
| Hydrogen peroxide scavenging activity | Between groups | 10.241 | 1 | 10.241 | 0.040 | **0.847** | No Significant difference |
|  | Within group | 2,053.048 | 8 | 256.631 |  |  |  |
|  | Total | 2,063.290 | 9 | - |  |  |  |
| Nitric oxide Scavenging activity | Between groups | 412.292 | 1 | 412.292 | 5.526 | **0.047** | Significantly low |
|  | Within group | 596.914 | 8 | 74.614 |  |  |  |
|  | Total | 1,009.206 | 9 | - |  |  |  |
| Reducing Power assay | Between groups | 0.045 | 1 | 0.045 | 4.063 | **0.079** | No Significant difference |
|  | Within group | 0.088 | 8 | 0.011 |  |  |  |
|  | Total | 0.133 | 9 | - |  |  |  |
| Cytotoxicity on HOS cell lines | Between groups | 28,429.083 | 6 | 4,738.181 | 4,273.471 | **0.000** | Significant differences in inhibition levels of different test concentrations |
|  | Within group | 15.522 | 14 | 1.109 |  |  |  |
|  | Total | 28,444.605 | 20 | - |  |  |  |

**Fig. S1** UV-Vis spectrum of the synthesized silver nanoparticles synthesized with the leaf

extract of *Rumex acetosa*.


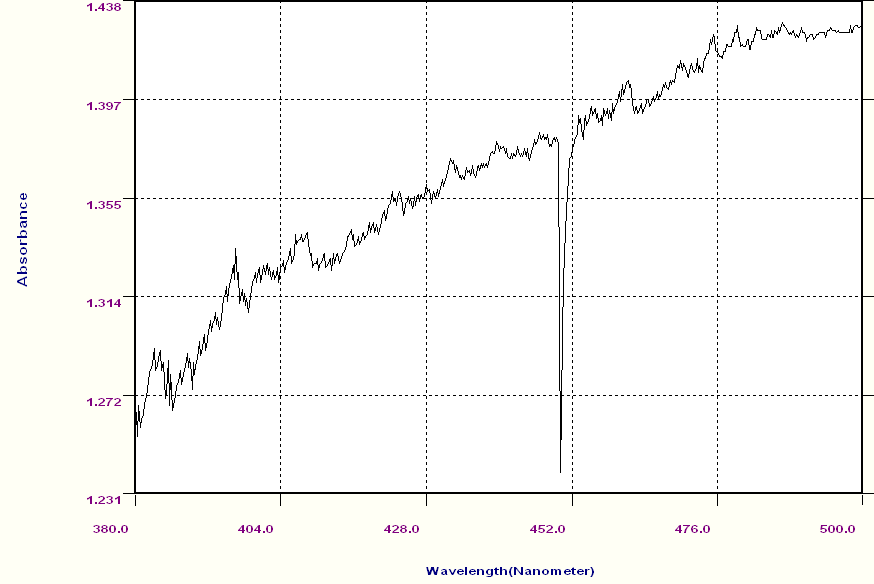


**Fig. S2** FT-IR Spectrum of synthesized silver nanoparticles with the extract of *Rumex acetosa*.

**
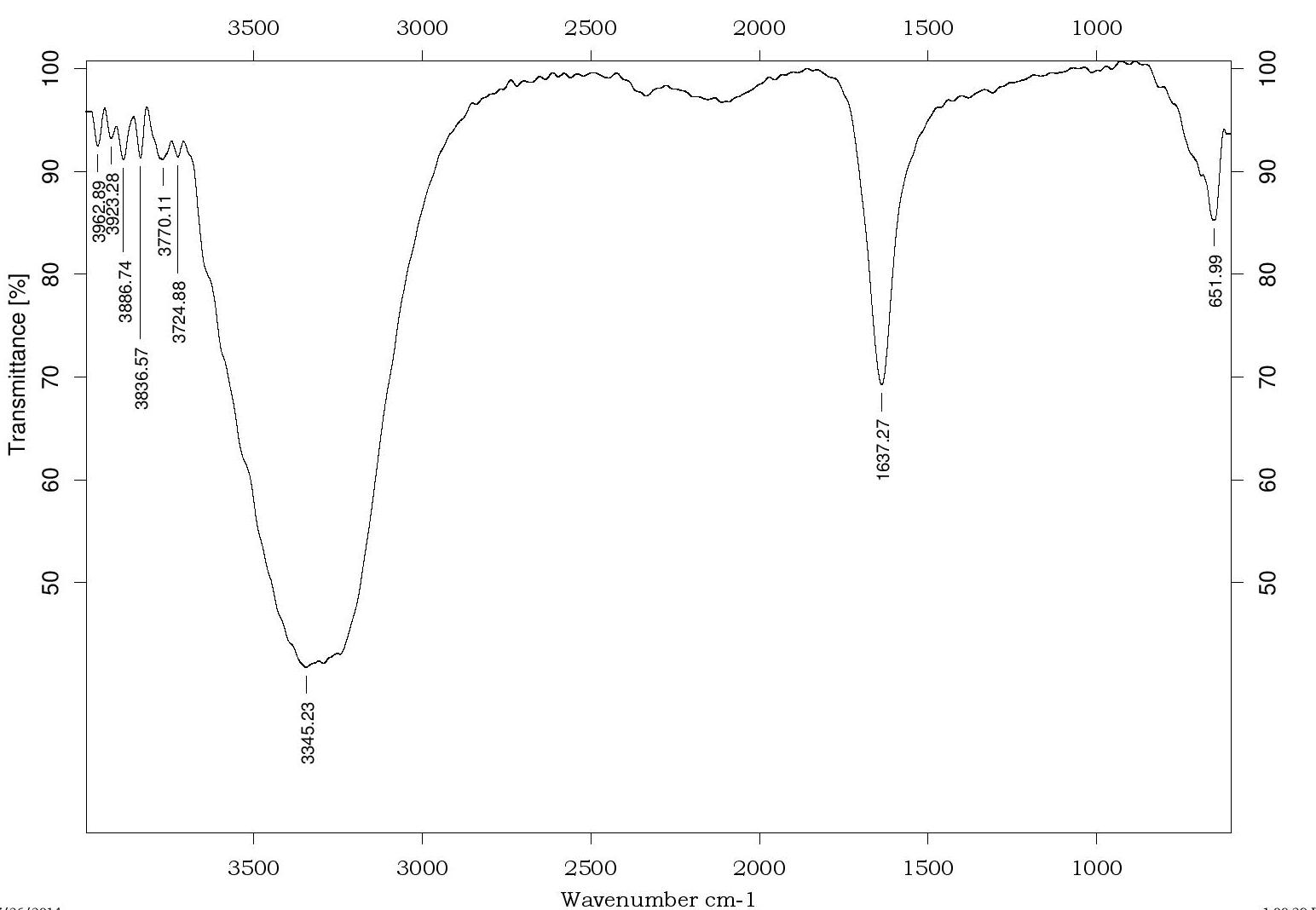
**

**Fig. S3** Transmission electron microscopic view of the synthesized silver nanoparticles with the

leaf extract of *R. acetosa*


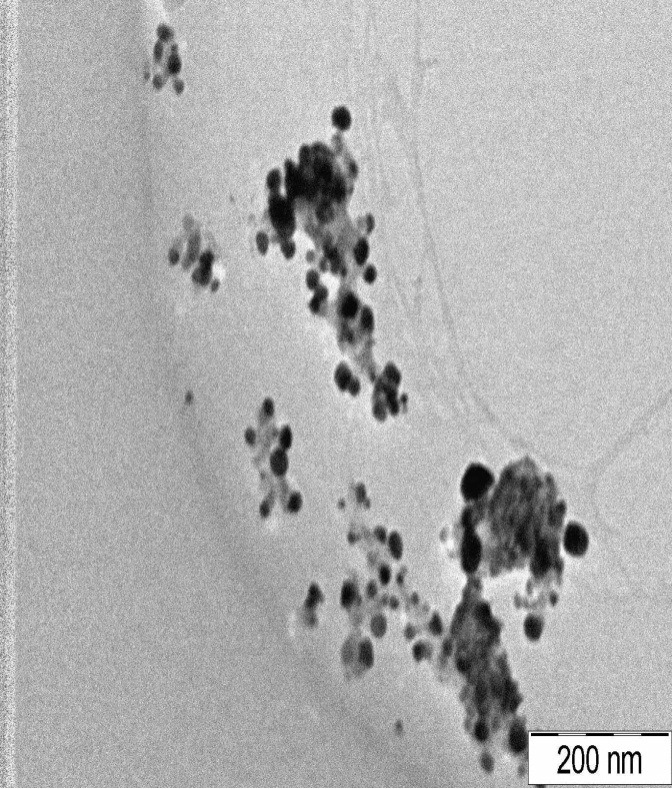


**Fig. S4** Size distribution of silver nanoparticles synthesized with the leaf extract of *R. acetosa*.

**
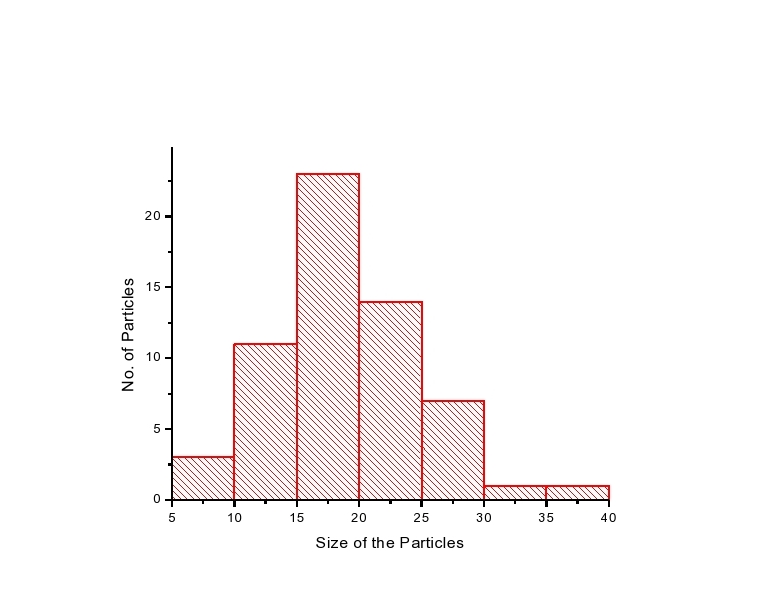
**

**Fig. S5a-S5b** HRTEM image of synthesized silver nanoparticles and Selected Area Electron

Diffraction (SAED) depicting crystalline nature of the sample.

Fig. S5a

**
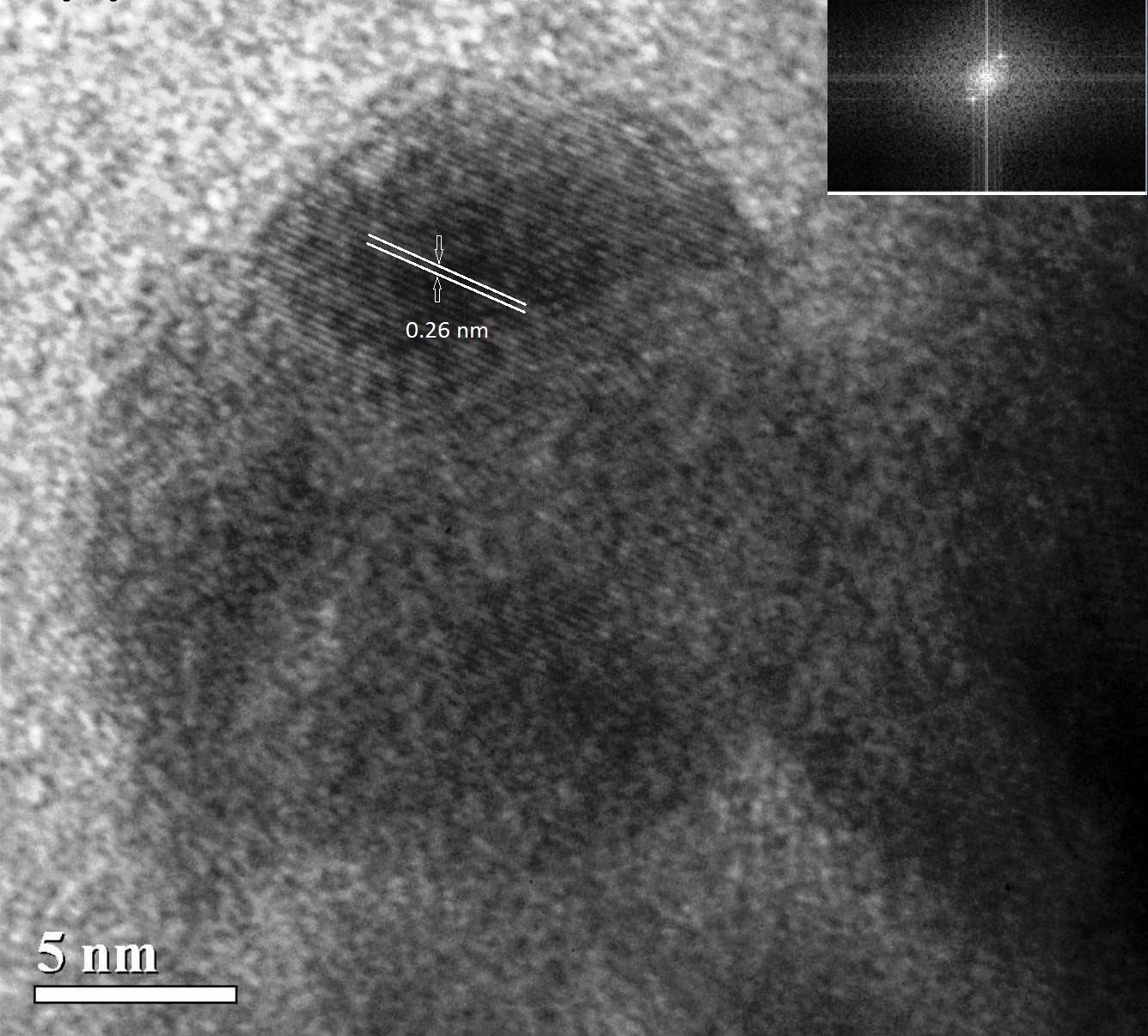
**

Fig. S5b

**
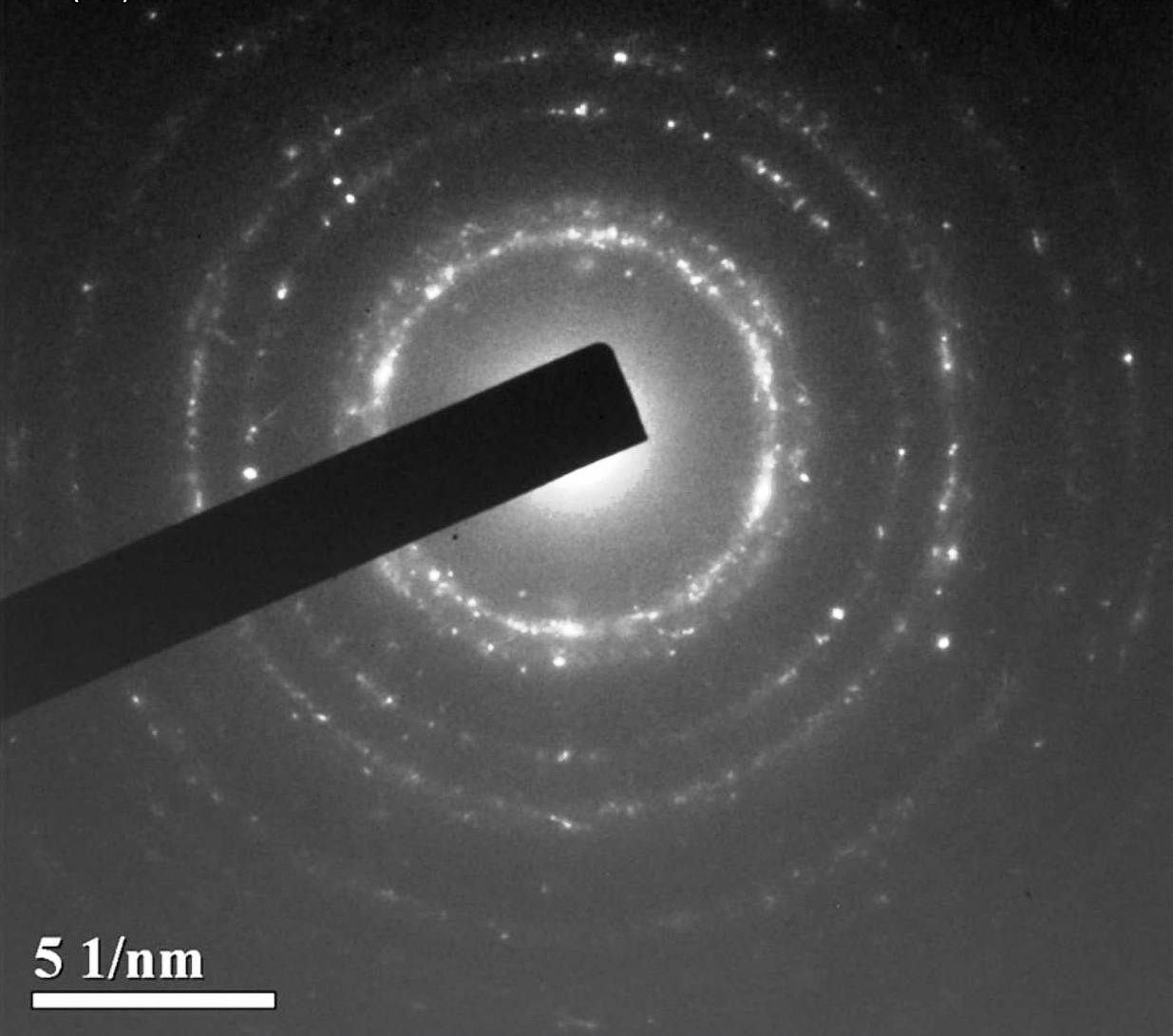
**

**Fig. S6a-6c** XPS – Ag, C1s and O1s peaks resolved.

**Fig. S6a**


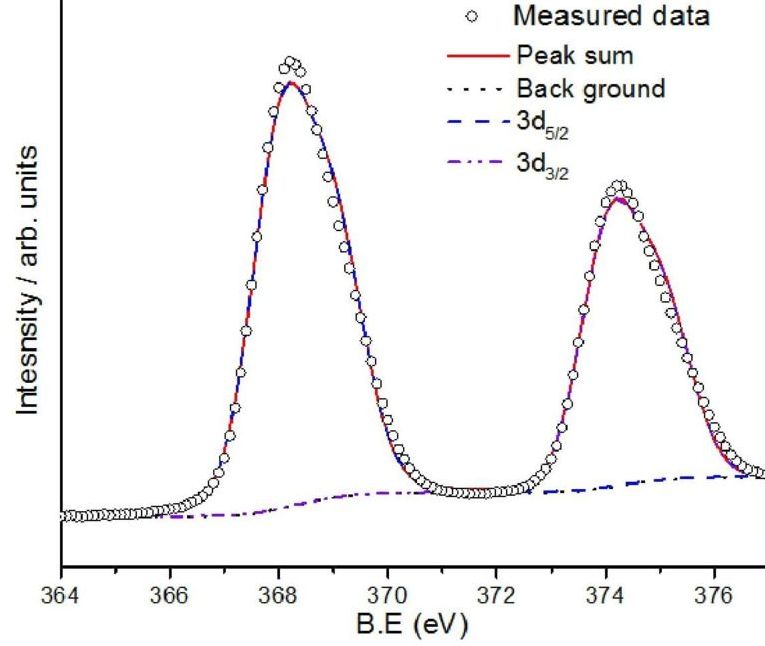


**Fig. S6b**


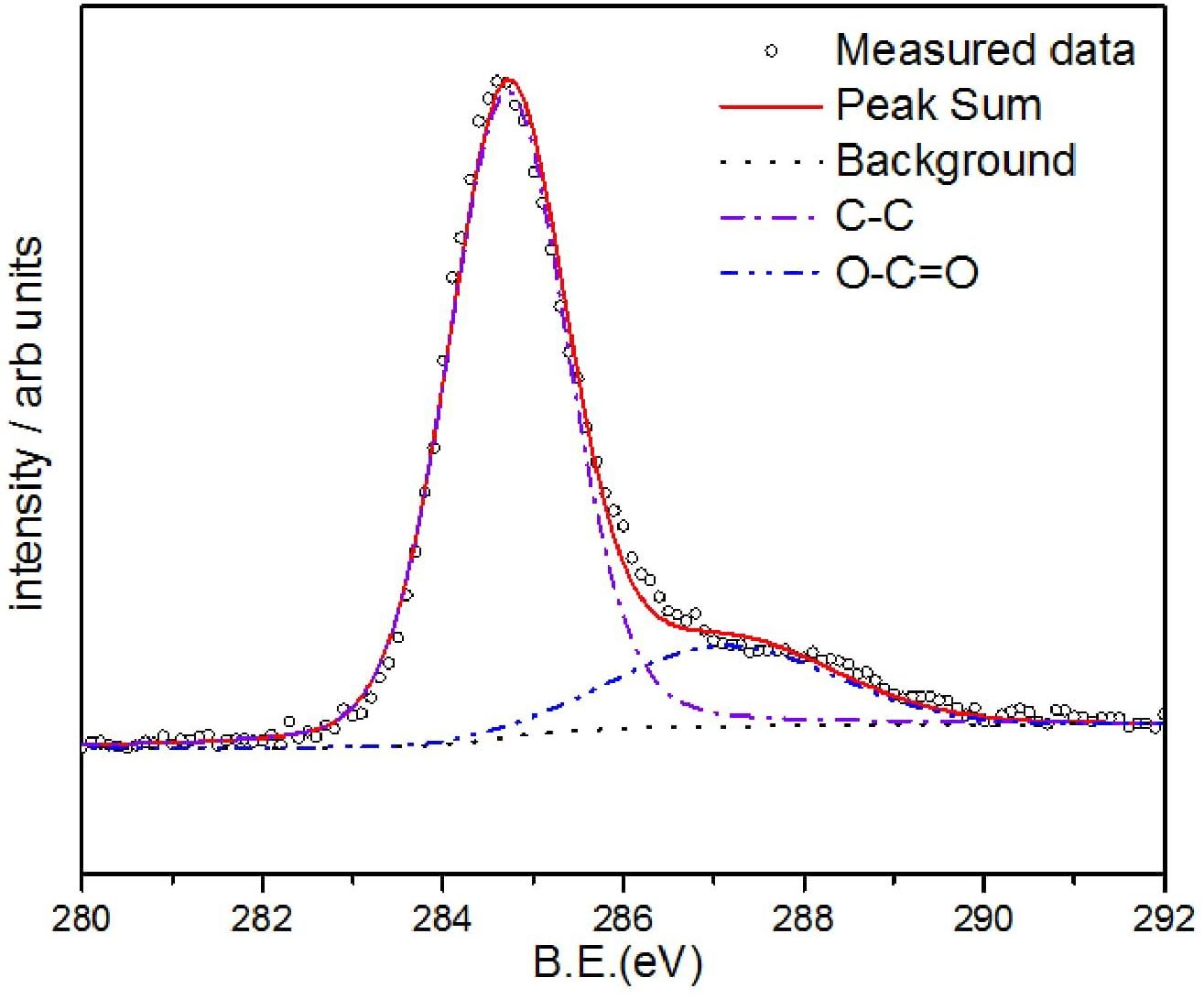


**Fig. S6c**


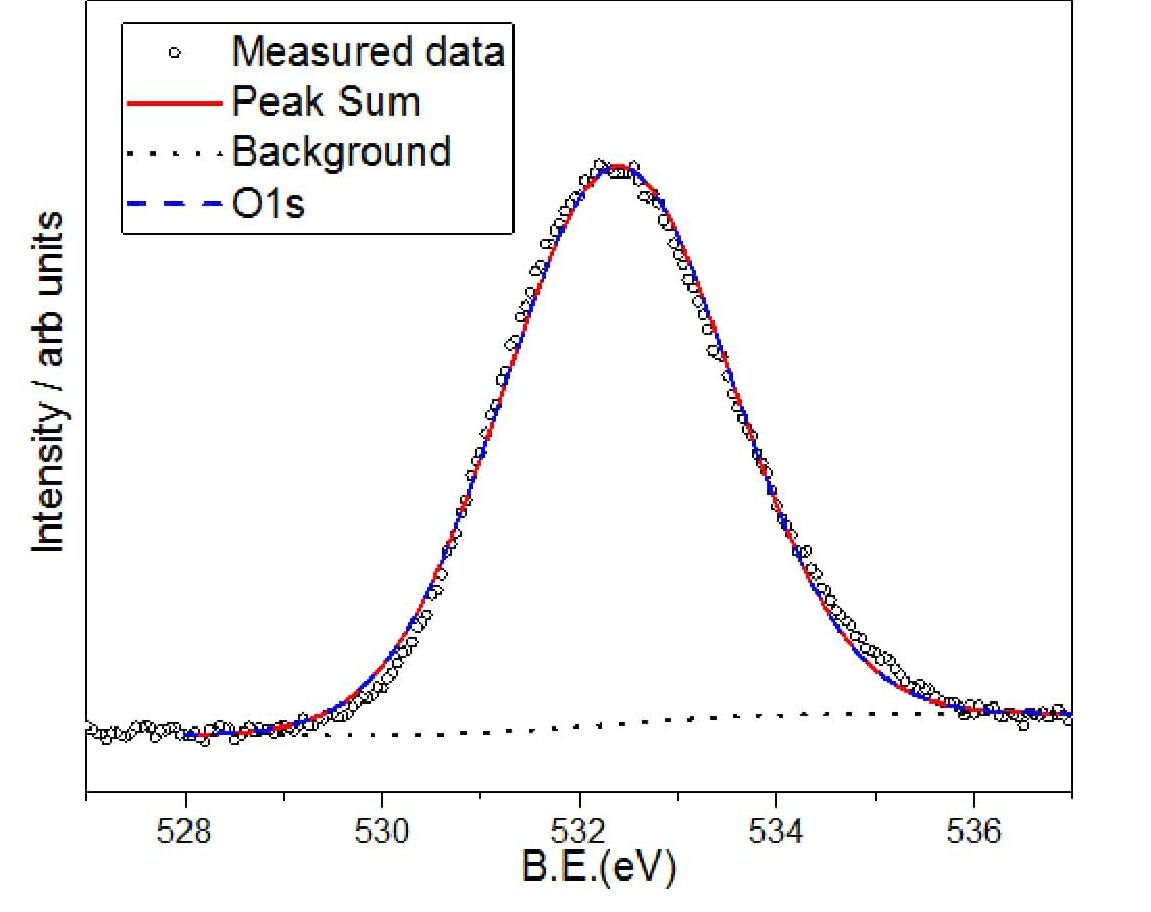


**Fig. S7** Micrograph of EDAX showing the different compositions of synthesized silver

nanoparticles with the leaf extract of *R. acetosa*.

**
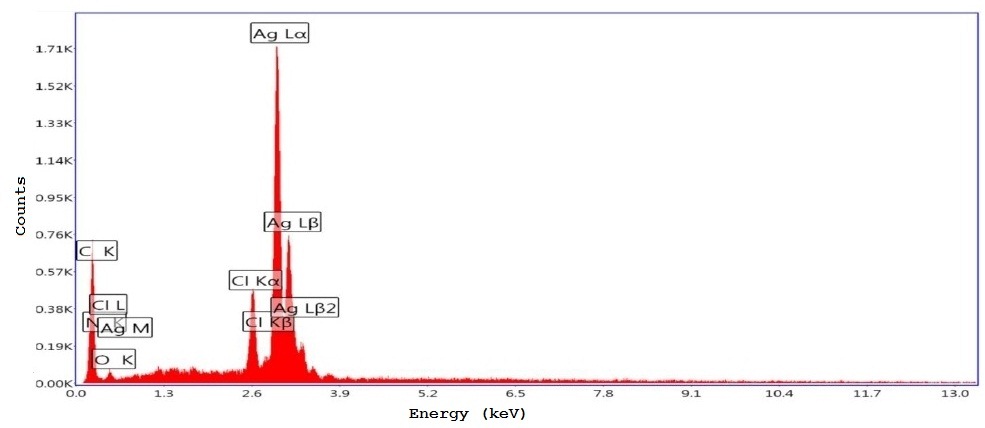
**

**Fig. S8** DPPH scavenging activity of synthesized Ag NPs with *R. acetosa* leaf extract, in

comparison with ascorbic acid


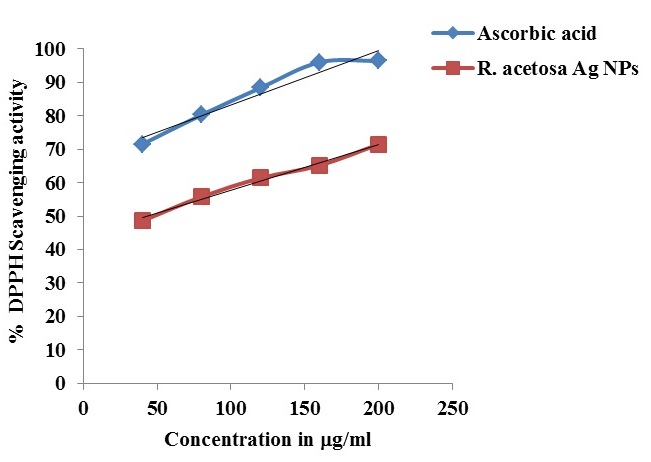


**Fig. S9** Hydrogen peroxide scavenging activity of the synthesized Ag NPs synthesized with *R.*

*acetosa* leaf extract in comparison with ascorbic acid as standard.


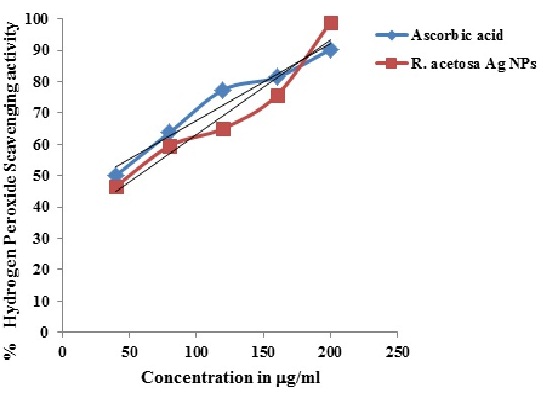


**Fig. S10** Reducing power assay to evaluate the activity of Ag NPs synthesized with *R. acetosa*

leaf extract in comparison with ascorbic acid as standard.

**
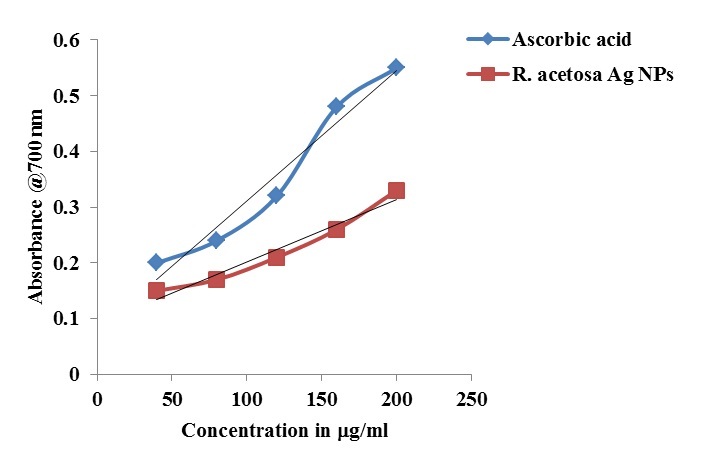
**

**Fig. S11** Nitric oxide scavenging activity of AgNPs synthesized with *R. acetosa* leaf extract in

comparison with ascorbic acid standard


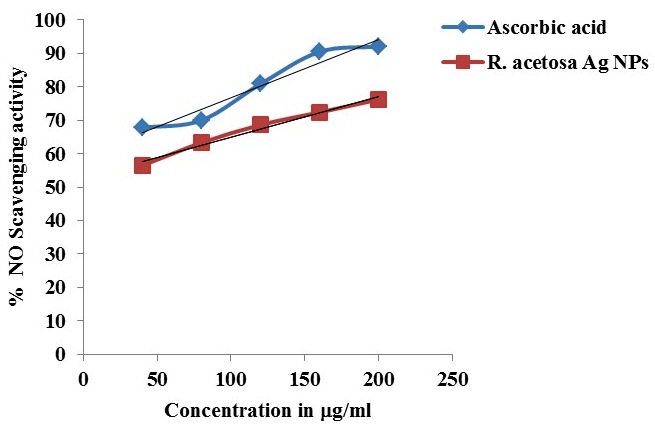

Supplement: Supplementary file 1 — Supplementary Information [file 41598_2017_11853_MOESM1_ESM.doc]
